# Association of procedure length on outcomes and adverse events of endoscopic retrograde cholangiopancreatography

**DOI:** 10.1093/gastro/gou009

**Published:** 2014-03-12

**Authors:** Paresh P. Mehta, Madhusudhan R. Sanaka, Mansour A. Parsi, Mazen J. Albeldawi, John A. Dumot, Rocio Lopez, Gregory Zuccaro, John J. Vargo

**Affiliations:** ^1^Digestive Disease Institute, Department of Gastroenterology and Hepatology, Cleveland Clinic, Cleveland OH, USA and ^2^Department of Quantitative Health Sciences, Cleveland Clinic, Cleveland, OH, USA

**Keywords:** endoscopic retrograde cholangiopancreatography (ERCP), duration of procedure, adverse events, pancreatitis

## Abstract

**Objective**: The aims of this study were to determine the effects of length of procedure on endoscopic retrograde cholangiopancreatography (ERCP) outcomes and adverse events.

**Methods**: All ERCP procedures, performed by experienced advanced endoscopists, in patients without prior papillary intervention from 2006 to 2008 were reviewed. Procedures were arbitrarily divided into two groups: shorter procedures (SP), with a duration shorter than the overall mean procedure length, and longer procedures (LP), with a duration longer than overall mean procedure length. Length of procedure was defined as the time from endoscope insertion to endoscope removal.

**Results**: Two hundred and ninety-five procedures were included in the analysis. Mean procedure length was 45.6 ± 30.1 min. One hundred and seventy-seven procedures (60%) were SP and 118 (40%) were LP. There were no differences between the groups with regard to patients’ ages, genders, race, or trainee participation. SP cases were more likely to be biliary vs pancreatic or bi-ductal evaluations (*P* = 0.03). LP had significantly higher complexity scores (34% with >3 vs 13%; *P* = 0.046) and were more likely to require pre-cut papillotomy (39% vs 15%; *P < *0.001). There was no significant difference between the groups in overall completion rates (91.5% LP vs 96% SP; *P* = 0.10) or adverse events (10.2% LP vs 6.2% SP; P = 0.21). However, LP cases were associated with higher rates of post-ERCP bleeding (4.2% vs 0.6%; *P* = 0.029).

**Conclusion**: There was no significant difference in outcomes or overall adverse events between shorter and longer ERCP procedures. However, longer procedures were associated with higher procedure complexity, higher utilization of pre-cut technique, and increased risk of bleeding.

## INTRODUCTION

Endoscopic retrograde cholangiopancreatography (ERCP) is one of the most technically demanding and highest-risk procedures performed by gastroenterologists [1]. Adverse events of ERCP include pancreatitis, bleeding, infection, perforation and sedation-related cardiopulmonary events. The literature focusing on patient-, procedure- and operator-related factors that are associated with outcomes in ERCP is vast [2–6]. Procedure-related factors such as multiple cannulation attempts, contrast injection into pancreatic duct, pancreatic brush cytology, minor papilla sphincterotomy and trainee involvement are associated with higher numbers of adverse events [7–8].

There is limited data evaluating the impact of length of ERCP procedures on outcomes and adverse events. Difficulty with cannulation and complex interventions required to accomplish the intended therapeutic goal may lead to prolonged procedure duration. These factors in turn could lead to poorer outcomes and more adverse events. The aims of this study were to determine the impact of ERCP procedure duration on outcomes and numbers of adverse events.

## METHODS

This study was approved by the Institutional Review Board of the Cleveland Clinic. We reviewed all ERCP procedures performed at our institution by experienced therapeutic endoscopists from November 2006 to November 2008. An experienced endoscopist was defined as one who has performed at least 500 ERCPs as an attending physician. This level of previous ERCP experience was used in order to assure some degree of uniformity in operator experience [2]. Exclusion criteria included patients with any previous papillary intervention, such as papillotomy, papillectomy, and stent placement. Patients with inadequate documentation of procedure durations—such as data on endoscope insertion time and endoscope removal time—were also excluded. Procedure length was defined as the time in minutes from endoscope insertion to its removal from the patient. Successful ductal cannulation was defined as achieving deep cannulation of the desired duct (biliary or pancreatic duct) based on procedure indication. Successful procedure completion was defined as achieving the desired therapeutic goal during the ERCP (e.g. complete removal of stones, successful stenting of stricture). ERCP complexity scores were defined as standard (grade 1), advanced (grade 2), and tertiary (grade 3), similar to the definitions proposed by the multi-society task force guidelines [1, 2]. ERCP complexity scores were estimated from the procedure reports and interventions performed. Based on the overall mean procedure duration of the entire cohort, procedures were divided for analysis into shorter procedures, SP (procedure duration lower than overall mean) and longer procedures, LP (procedure duration greater than overall mean). Post-procedure recovery time was defined as the duration in minutes from the end of procedure to the time of discharge from the endoscopy unit.

The electronic medical records and endoscopic reports of all included patients were reviewed. Adverse events were defined by using the American Society of Gastrointestinal Endoscopy workshop definitions [9]. Each patient’s electronic medical record was reviewed for post-ERCP hospital admissions, full laboratory analysis (complete blood count; amylase, lipase, liver function tests and blood cultures), discharge summaries, follow-up office visits, and any post-procedure patient–physician telephone contacts.

Descriptive statistics were computed for all variables. These include means, standard deviations and percentiles for continuous variables and frequencies and percentages for categorical factors. Subjects were divided into two groups, based on overall mean procedure duration (<45 min = SP vs ≥45 min = LP). Univariate analysis was performed to assess factors associated with longer procedure time. Student’s *t-*tests or the Wilcoxan rank sum tests were used to compare continuous variables and Pearson’s chi-squared tests were used for categorical variables. Receiver operating characteristics (ROC) analysis was performed to assess whether a cut-off point for procedure duration could be identified, for differentiating between complete and incomplete procedures. In addition, a multivariable logistic regression analysis was performed to evaluate adjustments for other factors, such as complexity score and type of procedure. An automated, stepwise, variable selection method, performed on 1000 bootstrap samples, was used to choose the final models; all variables were considered for inclusion and the areas under the ROC curves were estimated for combinations of the variables with highest inclusion rates. A *P*-value <0.05 was considered statistically significant. All analyses were performed using SAS (version 9.2, The SAS Institute, Cary, NC) or R (version 2.12.1, The R Foundation for Statistical Computing, Vienna, Austria).

## RESULTS

Of a total of 1066 ERCPs reviewed, 295 procedures were included in the final analysis. Duration of procedure ranged between 5 and 200 minutes, with a mean procedure duration of 45.6 ± 30.1 minutes. Of the 295 procedures included, 177 (60%) were SP and 118 (40%) were LP. Patient demographics were similar in both the groups (Table 1).

**Table 1.** Patient demographics

Factor	SP (*n* = 177)	LP (*n* = 118)	*P*-value
Age (years)	57.7 ± 17.0	61.2 ± 15.5	0.076
Male (%)	77 (43.5)	61 (51.7)	0.17
Race (%)			0.59
Caucasian	148 (83.6)	95 (80.5)	
African-American	20 (11.3)	18 (15.3)	
Other	9 (5.1)	5 (4.2)	

Values presented as Mean ± SD with *t-*test or N (%) with Pearson's chi-squared test.

SP procedures were more likely to be biliary (89.1% SP vs 78% LP; Table 2), while bi-ductal evaluations were more commonly LP (6.3% SP vs 13.6% LP). In addition, LP had significantly higher complexity scores, with 34% having a score of 3, compared with only 13% of SP (P = 0.046). LP was also more likely to require use of pre-cut papillotomy (SP 39% vs 15% LP; *P* < 0.001) for ductal access. SP procedures were more likely to have a normal cholangiogram (23.7% SP vs 14.4% LP) or choledocholithiasis (23.7% SP vs 16.1% LP), while LP were likely to have distal biliary strictures (18.6% SP vs 32.2% LP). There was no significant difference between the groups with regard to trainee participation (41.2% SP vs 50.8% LP; *P* = 0.10), use of moderate sedation (41.8% SP vs 53.4% LP; *P* = 0.051), or total post-procedure recovery time (59.0 min SP vs 60.0 min LP; *P* = 0.54).

**Table 2.** Procedure characteristics

Factor	SP (*n* = 177)	LP (*n* = 118)	*P*-value
Biliary/pancreatic procedures^a^			*0.033*
Biliary	156 (89.1)	92 (78.0)	
Pancreatic	8 (4.6)	10 (8.5)	
Both	11 (6.3)	16 (13.6)	
Moderate sedation	74 (41.8)	63 (53.4)	0.051
Trainee participation	73 (41.2)	60 (50.8)	0.10
ERCP complexity score^a^			*<0.001*
1	92 (52.3)	24 (20.3)	
2	61 (34.7)	54 (45.8)	
3	23 (13.1)	40 (33.9)	
Procedure findings			*0.046*
Normal	42 (23.7)	17 (14.4)	
Distal biliary stricture	33 (18.6)	38 (32.2)	
Choledocholithiasis	42 (23.7)	19 (16.1)	
PSC	8 (4.5)	6 (5.1)	
Ampullary stenosis	13 (7.3)	7 (5.9)	
Other	39 (22.0)	31 (26.3)	
Length of procedure (min)	25.0 [20.0, 35.0]	62.0 [55.0, 85.0]	–
Recovery time (min)^a^	59.0 [38.0, 78.0]	60.0 [39.0, 80.0]	0.54

^a^Data not available for all subjects. Missing values: biliary/pancreatic procedure = 2; ERCP complexity score = 1; recovery time (min) = 1.

Values presented as median [P25, P75] with Wilcoxon rank sum test or *n* (%) with Wilcoxon rank sum test for complexity score and Pearson's chi-squared test otherwise.

**Figure 1.**
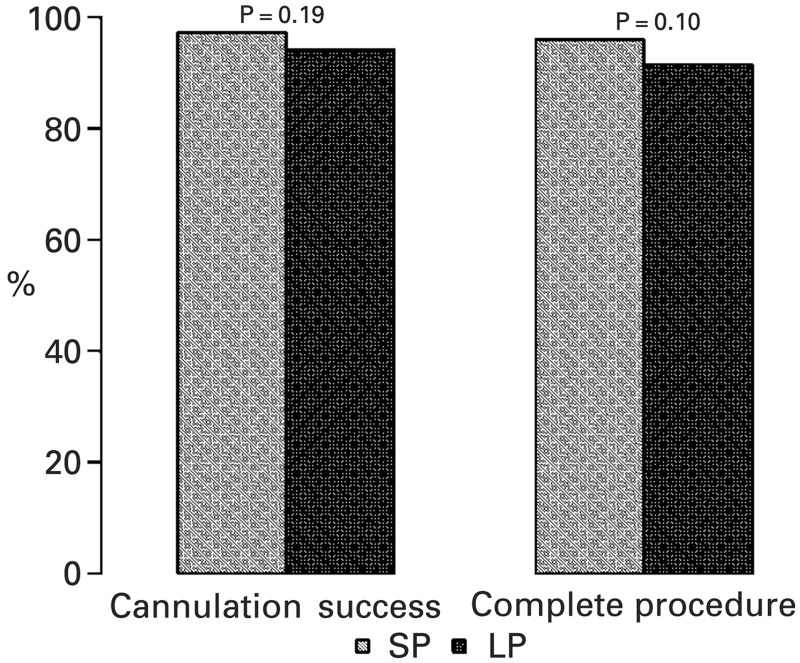
Deep duct cannulation success and procedure completion rates in SP vs LP.

**Figure 2.**
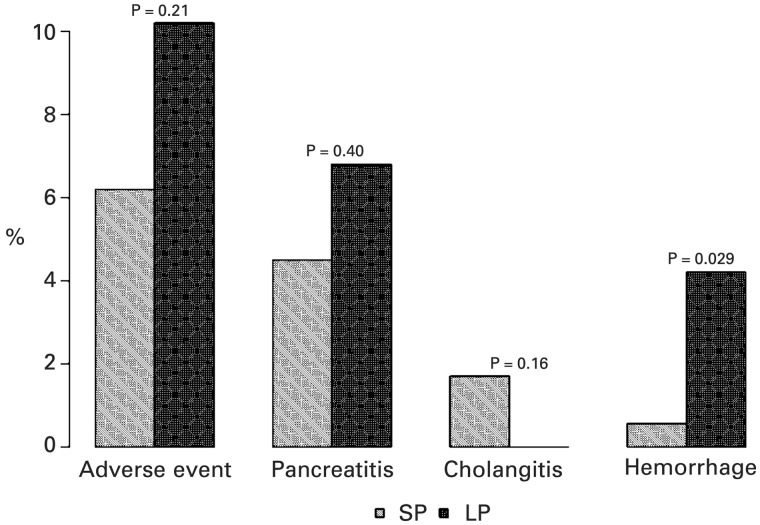
Overall and individual adverse events in SP vs LP.

Successful deep ductal cannulation rates (97.2% SP vs 94.1% LP; *P* = 0.19), procedure completion rates (96.0% SP vs 91.5% LP; *P* = 0.10) (Figure 1), and overall adverse events (6.2% SP vs 10.2% LP; *P* = 0.21) were similar in the two groups (Table 3); however, when adverse events were evaluated individually, there was a significantly higher rate of post-ERCP bleeding in the LP group (0.56% SP vs 5.0% LP; *P* = 0.029) (Figure 2).

**Table 3.** ERCP outcomes

Factor	SP (*n* = 177)	LP (*n* = 118)	*P*-value
Deep cannulation success	172 (97.2)	111 (94.1)	0.19
Complete procedure	170 (96.0)	108 (91.5)	0.10
Pre-cut papillotomy	27 (15.3)	46 (39.0)	*<0.001*
Sphincterotomy	132 (74.6)	76 (64.4)	0.061
Any pancreatitis	8 (4.5)	8 (6.8)	0.40
Any cholangitis	3 (1.7)	0 (0.0)	0.16
Any hemorrhage	1 (0.56)	5 (4.2)	*0.029*
Perforation	0 (0.0)	0 (0.0)	
Death	0 (0.0)	0 (0.0)	
Immediate or late adverse events^a^			0.47
No adverse events	165 (93.8)	106 (89.8)	
Immediate	1 (0.57)	1 (0.85)	
Late	10 (5.7)	11 (9.3)	

^a^Data not available for all subjects. Missing values: immediate or late adverse event = 1.

Values presented as *n* (%) with Wilcoxon rank sum tests for complication severity and Pearson's chi-squared test otherwise.

Immediate adverse events = events occurring within14 days of the procedure

Late adverse events = events occurring greater than 14 days after procedure

Spearman’s correlation coefficient (rho) showed a significant correlation between higher ERCP complexity scores and total length of procedure [rho: 0.43; 95 CI (0.32–0.53); *P* ≤ 0.001]. Length of procedure was not found to have good accuracy for prediction of either cannulation success [AUC (95% CI): 0.65 (0.49, 0.79)] or procedure completion rates [0.68 (0.56, 0.80)].

## DISCUSSION

To the best of our knowledge this is the first study to directly investigate the influence of ERCP procedure length on adverse events, deep duct cannulation success and procedure completion rates. We evaluated procedure length as a dichotomous variable, SP vs LP. There was no significant difference in overall adverse events, successful ductal cannulation rates or procedure completion rates between the two groups. However, LP was associated with higher incidence of post-procedure bleeding rates compared with SP.

Our study confirms the results of previous studies: that bi-ductal evaluations, higher ERCP complexity scores, and implementation of pre-cut techniques were more often observed for longer ERCP procedures [10]. We also found that longer procedures were associated with a significantly higher rate of post-ERCP bleeding, compared with shorter ones, despite similar rates of sphincterotomy. A possible explanation of the increased bleeding rates may be the higher use of pre-cut sphincterotomy techniques in the longer procedures, or that the length of ERCP was extended when bleeding occurred, in order to achieve hemostasis. In our study, the adverse events in the two groups—including post-ERCP bleeding rates—were low and are similar to rates previously reported in the literature [11].

Theoretically, longer procedures should fall into two groups: procedures with higher complexity and unsuccessful procedures. Higher complexity procedures would require a higher level of technical skill and therefore take longer. Unsuccessful procedures are usually prolonged, since the endoscopist attempts different methods or techniques to accomplish the desired intervention (e.g. deep duct cannulation, removal of large stones, or placement of a stent) [12–14]. We did note a trend towards more LPs being performed under moderate conscious sedation, rather than monitored anesthesia, although this was not statistically significant. Moderate sedation could potentially have led to longer procedure duration.

Although our study provides new information about the influence of procedure length on ERCP outcomes, there are some key limitations to this study, including its retrospective design. This study design may underestimate the rate of adverse events—such as post ERCP pancreatitis—when such events are not reported to the primary endoscopist or when the patients present to other medical centers for their treatment. Our study might be underpowered for detection of the differences in outcomes and adverse events. This study was performed at a tertiary care referral center, which might have led to possible selection and referral bias. However, there are several strengths in our study: it included only patients without previous papillary intervention, in order to avoid a spuriously high number of shorter procedures; only ERCPs performed by experienced endoscopists were included in the study and also trainee participation was similar in both the groups.

In conclusion, the length of ERCP procedure does not seem to influence successful deep ductal cannulation rate, procedure completion rate or overall adverse events. Higher complexity scores, bi-ductal evaluations, and use of pre-cut techniques may lead to longer ERCP procedures. Longer procedures might be associated with higher risk of post-procedure bleeding. Prospective studies are needed to validate these findings.

**Conflict of interest:** none declared.
